# Effect of population based tobacco cessation treatment in India

**DOI:** 10.6026/973206300221828

**Published:** 2026-03-31

**Authors:** Ruchi Mitra, Arpita Rai, Sarani Sagen Dahanga, Sandeep Kumar, Amit Kumar

**Affiliations:** 1 Department of Dentistry, Phulo Jhano Medical College and Hospital, Dumka-814110 Jharkhand, India; 2 Department of Oral Medicine and Radiology, Dental Institute, RIMS, Ranchi-834009, Jharkhand, India; 3 Department of Obstetrics and Gynaecology, Medinirai Medical College and Hospital, Palamu, Jharkhand-822118 India; 4 Department of Public Health Dentistry, Dental Institute, RIMS, Ranchi-834009, Jharkhand, India; 5 Department of Dentistry, Senior Dentist, Sadar Hospital, Dumka, Jharkhand, India

**Keywords:** Tobacco cessation, quit rate, behavior counselling, pharmacotherapy

## Abstract

Tobacco use remains a leading preventable cause of morbidity and mortality globally. The state of Jharkhand faces challenges because multiple forms of tobacco are in widespread use and limited resources are available for tobacco control. There is paucity of information related to the effectiveness of community-based tobacco cessation intervention and oral cancer screening program in Jharkhand therefore there is need for the present study. This quasi-experimental study in Ranchi district, Jharkhand, evaluated the effectiveness of an integrated, population-based tobacco cessation intervention implemented in Ayushman Aarogya Mandirs. The intervention comprised pharmacotherapy, behavioural counselling, and capacity building of community health workers. After one year, 657 of 4429 participants achieved cessation, representing a quit rate of 15.03% indicating the effectiveness of involving community health workers at grass root level. Findings demonstrate that combination therapy is more effective than behaviour counselling alone. Thus, we show the value of integrated cessation strategies in public health interventions, particularly in settings with a high burden of tobacco use.

## Background:

Tobacco use remains one of the leading preventable causes of disease and death worldwide, contributing to numerous chronic diseases, including cancer, respiratory conditions, cardiovascular diseases, and diabetes. The World Health Organisation (WHO) reports that over 8 million people die every year due to tobacco use, with the vast majority of deaths occurring in low and middle-income countries, where tobacco use patterns are often unregulated, and prevention measures are insufficient [1]. The direct link between tobacco consumption and various types of cancers, particularly oral cancer, is well established. Oral cancers, including cancers of the mouth, tongue, pharynx, and larynx, account for a significant proportion of cancer-related deaths in many countries, particularly in regions with high rates of tobacco use [2, 3]. Oral cancer is a major public health challenge in India, accounting for 30% to 40% of all cancers. India has one of the highest rates of oral cancer in the world, with tobacco use being the primary cause. According to the World Health Organisation (WHO), tobacco cessation interventions can lead to significant health improvements, including a reduction in the risk of developing cancers, especially oral cancer, which is directly linked to tobacco consumption. Moreover, cessation efforts not only benefit individuals who quit tobacco but also positively impact public health at the population level by reducing the overall incidence of tobacco-related diseases [4]. Jharkhand, located in eastern India, is a state with a high burden of tobacco-related diseases. The state's population, particularly in rural and semi-urban areas like Ranchi, exhibits high rates of tobacco consumption.

Jharkhand's tobacco use statistics surpass the national average, with a significant portion of its adult population engaged in smokeless tobacco consumption, such as chewing Gutkha, Khaini and Paan. The prevalence of tobacco use is higher than the national average in Jharkhand, with approximately 38.9% of the adults aged 15 or above using tobacco in any form. Around 5500 cancer-related deaths are reported in Jharkhand every year [5]. Ranchi, the capital city of Jharkhand, serves as a critical urban centre in the region but still faces similar public health challenges as its rural counterparts. The state of Jharkhand faces particular challenges because multiple forms of tobacco are in widespread use, and limited resources are available for tobacco control. Moreover, there is a paucity of information related to the effectiveness of community-based tobacco cessation interventions in Jharkhand. Therefore, it was of interest to assess the impact of population-based integrated tobacco cessation treatment in Ranchi district, Jharkhand.

## Materials and Methods:

## Study design:

The present study comprised a quasi-experimental study with a dissemination-implementation research framework embedded. The intervention was delivered in one of the blocks of Ranchi district Jharkhand. The intervention aligned with the objectives of the National Tobacco Control Programme (NTCP) and the Ayushman Bharat Health and Wellness Centre framework. Implementation of an integrated tobacco cessation treatment was delivered. Ethical approval for the study was obtained from the Institutional Ethics Committee and Departmental Research Committee, RIMS, Ranchi. Written informed consent was obtained from all participants before data collection. The intervention was conducted at block level. Ranchi has 304 HWCs (Health and Wellness Centres), of which Kanke block has 10, all of which were functional during the study period. We are involved with the HWC presently known as Ayushman Aarogya Mandir (AAM). The HWC comprises 2-3 Accredited Social Health Activists (ASHA) workers, known as Sahiya in Jharkhand. They are trained under the National Rural Health Mission programme of the Government of India. They work as an interface between the community and the public health system. Their main work is to raise awareness of health and its social determinants, complete the CBAC (Community-based Assessment Checklist), and mobilise patients to the HWC for treatment.

## Sample size and sampling technique:

A convenience sampling methodology was used as we included all patients visiting AAM Kanke during the one-year period between July 2023 and June 2024 who had tobacco habits, and they were followed up for a further one year until June 2025.

## Sample selection:

All the patients visiting the AAM Kanke study block were screened for tobacco habits and oral cancer by the CHO. The Sahiyasmobilised patients after complete the Community-based Assessment Checklist (CBAC).

## Inclusion criteria:

[1] Patients with a habit of using tobacco in either form, as smokeless and/or smoked tobacco products, in the HWC Kanke blocks.

[2] Individuals who were willing to participate

[3] Individuals in the age group 15-65 years.

## Exclusion criteria:

[1] Patients with comorbidities like cardiovascular problems, such as myocardial infarction, Heart attack, and severe neurological problems

[2] Pregnant ladies

[3] Patients having other substance abuse like heroin, Ganja, etc.

## Methodology:

Before the start of the intervention for promotion and advocacy of the study, the following programs were conducted:

## Screening camps:

## Intervention components:

[1] Training of CHOs and Sahiyas at six-month intervals

[2] Behavioral counselling using the 5A's framework(Role play model was used to teach the CHO)

[3] Assessment of nicotine dependence using the Fagerström Test

[4] Pharmacotherapy, including Nicotine Replacement Therapy (NRT)

[5] Non-Nicotine Replacement Therapy (NNRT), prescribed via e-Sanjeevani teleconsultation

[6] Distribution of the IEC (Information, education and communication) pamphlets

## Data compilation and presentation:

The obtained data were compiled systematically. A master table was prepared, and the dataset was subdivided and distributed meaningfully, with the results presented as individual tables and graphs.

## Bias control and methodological rigour:

Confounding was addressed through stratified analysis by age and gender during the interpretation of results.

## Statistical analysis:

Data were analysed using SPSS version 27.0 (IBM Corp., Chicago, IL, USA) and Jamovi version 2.3. Descriptive statistics were expressed as mean ± standard deviation for continuous variables and frequency (percentage) for categorical variables. Normality was assessed using the Kolmogorov-Smirnov test. Between-group comparisons were performed using the unpaired Student's t-test for normally distributed variables and the chi-square test for categorical variables. A p-value <0.05 was considered statistically significant.

## Results:

Tobacco use and oral cancer screening was conducted across10 AAM (Ayushman Arogya Mandir) of Kanke study block (Two AAM of Kanke were non-functional). Of these, 4,429 tobacco users were found; 58 patients refused to accept the treatment. Therefore, a total of 4371 patients were given tobacco cessation treatment and followed up for one year. Table 1 (see PDF) presents the socio-demographic profile with mean age 34.8±1.3 and majority of study population being males (60.03%). Most of the study participants had completed their higher secondary school and were no government employed with family income between Rs 9308 to Rs 27882. Overall, the baseline socio-demographic characteristics and tobacco use status were statistically non-significant and FTND scores (p-values shown as 0.082, 0.112 and 0.093, respectively. The distribution of the variable did not significantly differ from a normal distribution on normality tests. Table 2 (see PDF) presents abstinence rates at 12 months according to the type of cessation intervention received. Among participants receiving behavioural counselling alone (BC), 283 out of 2,987 participants achieved abstinence at 12 months, corresponding to a quit rate of 9.47%. In the BC + Nicotine Replacement Therapy (NRT) group, 352 out of 1,323 participants achieved abstinence, yielding a quit rate of 26.6%. In the BC + Non-Nicotine Replacement Therapy (NNRT) group, 22 out of 61 participants achieved abstinence, corresponding to a quit rate of 36.05%. Adjusted odds ratios indicated a statistically significant association between pharmacotherapy use and abstinence outcomes, with higher odds of abstinence observed in the pharmacotherapy-assisted groups compared with behavioural counselling alone (p < 0.05). Table 3 (see PDF) presents the results of Cox proportional hazards analysis evaluating time to relapse among participants receiving different cessation interventions in the study block. Among participants receiving behavioural counselling alone, 845 relapse events and 1,727 censored observations were recorded. In the BC + NRT group, 431 relapse events and 679 censored observations were observed, while in the BC + NNRT group, 19 relapse events and 21 censored observations were recorded. Hazard ratios indicated a significantly lower hazard of relapse among participants receiving pharmacotherapy-assisted interventions. The hazard ratio for BC + NRT was 0.453 (95% CI: 0.245-0.816; p = 0.013), and for BC + NNRT was 0.397 (95% CI: 0.213-0.654; p = 0.001), compared with behavioural counselling alone. The mean time to relapse was 5.45 ± 3.61 months for behavioural counselling alone, 9.76 ± 4.33 months for BC + NRT and 10.44 ± 3.25 months for BC + NNRT. Figure 1 (see PDF) represents the quit rates of the study block. A total of 1,054 participants reported quitting tobacco at 3 months, corresponding to a quit rate of 24.14%. At 6 months, 892 participants had quit, yielding a quit rate of 20.4%. At 9 months, 813 participants were reported as quitters (18.7%) and at 12 months, 657 participants remained abstinent, corresponding to a quit rate of 15.03%. Figure 2 (see PDF) shows that out of 4429 patients, 58 patients were reluctant to accept the treatment. There was a loss to follow up due to various reasons, and 2419 patients were lost. A total of 657 patients successfully quit tobacco in the present study.

## Discussion:

The present implementation and dissemination study was undertaken to evaluate the effectiveness of an integrated tobacco cessation treatment intervention delivered through routine primary healthcare services in a high-tobacco-burden setting of eastern India. This discussion interprets the findings in relation to the stated objectives, situates them within the context of national and international evidence, and explains the observed outcomes. The present study is the first community-based long-term study that focuses on involving the health care officers at the primary level to help in initiating quitting among the community residents. The predominance of smokeless tobacco products such as gutkha and khaini among study participants is consistent with national and regional epidemiological patterns. GATS (Global Adult Tobacco Survey) and NFHS (National Family Health Survey) data indicate that smokeless tobacco use is particularly prevalent in eastern and central India, driven by cultural acceptance, affordability, and misconceptions regarding relative safety compared with smoking [6, 7]. Similar results were found in a meta- analysis done in a tribal population, which reported an alarmingly high consumption of smokeless tobacco and cultural acceptability [19]. Previous Indian studies have reported lower quit rates among smokeless tobacco users than among smokers, often attributing this to lower perceived risk and limited availability of cessation resources tailored to smokeless products [4]. However, the present study demonstrated meaningful reductions in smokeless tobacco use following intervention. This finding aligns with emerging evidence that smokeless tobacco dependence is biologically similar to smoking- related dependence and responds to pharmacotherapy when combined with appropriate behavioural support [8]. A recent meta-analysis conducted on pharmacological interventions for smokeless tobacco reported that the pharmacological intervention methods are more effective than behavioural intervention alone [20]. Nicotine dependence emerged as a critical mediator of cessation success in the present study. Participants with low to moderate dependence were significantly more likely to achieve and maintain abstinence, while those with high dependence experienced higher relapse rates. This gradient is well documented in addiction literature and reflects neuro-adaptive changes associated with chronic nicotine exposure [10, 24]. Importantly, pharmacotherapy substantially reduced the negative impact of higher dependence on cessation outcomes. This observation supports findings from systematic reviews demonstrating that medication-assisted cessation approximately doubles the likelihood of successful quitting compared with behavioural support alone. In the Indian context, where pharmacotherapy has historically been underutilised in public sector settings, these findings provide strong justification for its wider adoption [16]. Sustained abstinence at 12 months is widely regarded as the most robust indicator of cessation success. The 12-month quit rate observed in the study block compares favourably with rates reported in Indian community-based cessation trials, which typically range from 5% to 12% [9]. Internationally, combined behavioural and pharmacological interventions have yielded quit rates of 15-25%, largely in high-income settings [15, 21]. Participants receiving nicotine replacement therapy or non-nicotine pharmacotherapy demonstrated significantly higher quit rates and longer relapse-free intervals than those receiving behavioural counselling alone. These findings are consistent with global evidence supporting the superiority of combined treatment approaches [11, 22 and 23]. Concerns regarding feasibility, adherence, and cost have often limited the use of pharmacotherapy in low-resource settings. However, the present study demonstrates that these barriers can be addressed through simplified protocols, dependency-based prescribing, and integration with routine follow-up. Similar conclusions have been drawn from WHO PEN (World Health Organisation Package of essential non-communicable disease) implementation studies, which emphasise the cost-effectiveness of essential NCD (Non-communicable disease) interventions at the primary care level [1, 13]. Relapse remains a major challenge in tobacco cessation, particularly in community-based programs with limited long-term follow-up. Participants receiving pharmacotherapy not only experienced fewer relapses but also relapsed later, indicating more stable cessation trajectories. Few Indian cessation studies have employed relapse analysis and most rely on point- prevalence measures [11, 18]. A substantial proportion of participants transitioned from precontemplation and contemplation to action and maintenance stages, reflecting internalised motivation rather than transient compliance. This pattern supports the applicability of the transtheoretical model of behaviour change in Indian community settings. Repeated counselling, motivational interviewing, and self-monitoring tools likely contributed to these behavioural shifts [12, 17]. Similar findings have been reported in Indian studies conducted in Kerala and Karnataka, where structured follow-up was associated with sustained behaviour change [14]. Similar findings were observed in an evidence-based systematic review worldwide that long term effectiveness of tobacco cessation involves a holistic approach [24]. The use of the teleconsultation e-sanjeevani platform was the first of its kind used for tobacco cessation in the present study. The finding of the present study suggests that community health officers can play an important role in tobacco cessation. A study reported that community led officers improved the smoking abstinence [25]. However there were no studies similar to our study which showed the effectiveness in both smoking and smokeless tobacco cessation. This was the newer approach to bridge the gap between primary and tertiary health centres and to integrate the two programs of NTCP and AAM tobacco services, which would be more efficient if they collaborated. Moreover, the patients had easy access and convenience to the pharmacotherapy and quitting was more possible due to community interaction. The limitation of the present study could be the self-reported abstinence of the patients.

## Conclusion:

Data provides strong, context-specific evidence that an integrated tobacco cessation treatment intervention delivered through primary healthcare can achieve sustained reductions in tobacco use. By embedding treatment within routine services and leveraging existing human resources, the intervention addresses key gaps in current tobacco control strategies. The findings have important implications for policy, program design, and future research in high-burden, resource-constrained settings and integration of health programs for better and sustained development.
